# S11-2 Co-production of a physical activity referral scheme in the German health care system

**DOI:** 10.1093/eurpub/ckad133.054

**Published:** 2023-09-11

**Authors:** Eriselda Mino, Sarah Klamroth, Anja Weissenfels, Inga Naber, Wolfgang Geidl, Peter Gelius, Karim Abu-Omar, Klaus Pfeifer

**Affiliations:** Department of Sport Science and Sport, Friedrich-Alexander-Universität Erlangen-Nürnberg, Germany; Department of Sport Science and Sport, Friedrich-Alexander-Universität Erlangen-Nürnberg, Germany; Department of Sport Science and Sport, Friedrich-Alexander-Universität Erlangen-Nürnberg, Germany; Department of Sport Science and Sport, Friedrich-Alexander-Universität Erlangen-Nürnberg, Germany; Department of Sport Science and Sport, Friedrich-Alexander-Universität Erlangen-Nürnberg, Germany; Department of Sport Science and Sport, Friedrich-Alexander-Universität Erlangen-Nürnberg, Germany; Department of Sport Science and Sport, Friedrich-Alexander-Universität Erlangen-Nürnberg, Germany; Department of Sport Science and Sport, Friedrich-Alexander-Universität Erlangen-Nürnberg, Germany

## Abstract

**Purpose:**

Physical activity referral schemes (PARS) have the potential to promote physical activity (PA) among the mostly inactive primary care population with non-communicable diseases (NCD). Despite significant expansion throughout Europe, a national scheme has not yet been established in Germany. Thus, the ongoing research project BewegtVersorgt aims to develop, implement, and evaluate a PARS to promote PA for persons with NCD within the German healthcare system.

**Methods:**

The PARS was developed using a co-production approach which integrated twelve organizations from different sectors within the healthcare system (health insurances, physicians, exercise professionals, patients’ representatives) and was guided by the research team. Initially, stakeholders’ ideas of a PARS were collected in individual interviews. These were followed by three co-production meetings where individual ideas and current evidence were combined, and different PARS models for the German setting were elaborated. Finally, all stakeholders agreed on one PARS for implementation as a regional model project.

**Results:**

The co-produced PARS starts with the primary care physician, as the gatekeeper in the German healthcare system, who offers 10 min PA advice to the patients who would benefit from PA participation. Afterward, the patients are referred to an exercise professional through a referral form. The exercise professional delivers the remaining central components of the intervention - initial assessment, six 1-hour individual PA counseling sessions spread throughout 12 weeks, final assessment, a progress report to the general practitioner, and one follow-up visit at six months. The intervention uses a patient-centered approach and motivational interviewing. The end goal is an adaptation of an active lifestyle and transfer to local physical activity opportunities.

**Conclusions:**

By using a co-production approach, the expertise of different stakeholders within the healthcare system as well as scientific findings could be integrated into the development process of the PARS. To which extent this participatory approach leads to greater acceptance of the new healthcare service among the implementing actors and positively influences their cooperation within the PARS will be evaluated during the implementation phase.

**Support/Funding Source:**

The study is funded by the Federal Ministry of Health based on a resolution of the German ‘Bundestag’ by the federal government.

